# Differential Properties of the Synaptogenic Activities of the Neurexin Ligands Neuroligin1 and LRRTM2

**DOI:** 10.3389/fnmol.2019.00269

**Published:** 2019-11-08

**Authors:** Sushma Dagar, Kurt Gottmann

**Affiliations:** Institute of Neuro- and Sensory Physiology, Medical Faculty, Heinrich Heine University Düsseldorf, Düsseldorf, Germany

**Keywords:** synaptic adhesion, synaptogenic activity, Neuroligin1, LRRTM2, N-cadherin

## Abstract

Synaptic cell adhesion molecules are well established to exhibit synaptogenic activity when overexpressed in target cells, indicating that they are involved in formation and functional maturation of synapses. The postsynaptic adhesion proteins Neuroligin1 and LRRTM2 both induce synaptic vesicle clusters in presynaptic axons *in vitro* by transsynaptically interacting with neurexins. In neurons, this is accompanied by the induction of glutamatergic, but not GABAergic synapses. Although the synaptogenic activity of Neuroligin1 has been well characterized, the properties of the synaptogenic activities of other synaptic adhesion molecules are largely unknown. In this paper, we now compared characteristics of the synaptogenic activities of Neuroligin1 and LRRTM2 upon overexpression in cultured mouse cortical neurons. Individual cortical neurons were transfected with Neuroligin1 and LRRTM2 expression plasmids, respectively, and synaptic vesicle clustering in contacting axons was examined by immunostaining for the vesicle membrane protein VAMP2. In immature neurons at 6–7 days *in vitro* (DIV) both Neuroligin1 and LRRTM2 exhibited strong synaptogenic activity. However, upon further neuronal differentiation only LRRTM2 retained significant synaptogenic activity at 12–13 DIV. A similar differential developmental maturation of the synaptogenic activities of Neuroligin1 and LRRTM2 was observed for the induction of glutamatergic synapses, which were detected by co-immunostaining for VGLUT1 and Homer1. Most interestingly, the synaptogenic activity of Neuroligin1 was strongly dependent on the expression and function of the synaptic adhesion molecule N-cadherin in immature neurons. In contrast, the synaptogenic activity of LRRTM2 was independent of N-cadherin expression and function in both immature (6–7 DIV) and more mature neurons (14–15 DIV). Taken together, our results with overexpression in cultured cortical neurons revealed striking differences in the properties of the synaptogenic activities of Neuroligin1 and LRRTM2, although both transsynaptically interact with presynaptic neurexins.

## Introduction

Synaptic cell adhesion molecules are thought to play crucial roles in synapse formation, in synapse function, and in synaptic plasticity (Siddiqui and Craig, 2011; Missler et al., 2012; Rudenko, 2017; Südhof, 2018). A characteristic feature of these transmembrane proteins is that they exhibit synaptogenic activity if overexpressed in non-neuronal cells as well as in neurons (Scheiffele et al., 2000; Biederer et al., 2002; Linnhoff et al., 2009). Expression of the synaptic adhesion molecule Neuroligin1 in non-neuronal cells or in neurons is well known to result in the formation of presynaptic vesicle clusters in contacting axons *in vitro* (Scheiffele et al., 2000; Sara et al., 2005; Stan et al., 2010).

Neuroligins are postsynaptic adhesion molecules with Neuroligin1 expressed selectively at excitatory synapses (Song et al., 1999; Krueger et al., 2012). They bind transsynaptically to presynaptic neurexins thus forming the heterophilic neurexin–neuroligin signaling system (Krueger et al., 2012; Südhof, 2017). This molecular interaction has been described to mediate the *in vitro* synaptogenic activity of postsynaptically overexpressed Neuroligin1 (Dean et al., 2003; Graf et al., 2004). Members of the LRRTM family of postsynaptic adhesion proteins (e.g., LRRTM2) also bind to presynaptic neurexins (de Wit et al., 2009; Ko et al., 2009; Siddiqui et al., 2010) thus forming a molecularly complex transsynaptic signaling system with neurexins as presynaptic hub proteins (Südhof, 2017). Similar to Neuroligin1, LRRTM2 has been described to exhibit synaptogenic activity in *in vitro* axon contact assays by interaction with presynaptic neurexins (de Wit et al., 2009; Ko et al., 2009; Siddiqui et al., 2010). In cultured hippocampal neurons, LRRTM2 overexpression led specifically to the formation of additional glutamatergic synapses without inducing GABAergic synapses (de Wit et al., 2009).

The synaptogenic activity of Neuroligin1 has been characterized in detail upon overexpression in cultured neurons. A strong influence of the developmental maturation of neurons *in vitro* has been described, with immature neurons associated with strong synaptogenic activity of Neuroligin1 (Wittenmayer et al., 2009; van Stegen et al., 2017). Furthermore, a strict dependence on the expression and function of the synaptic adhesion molecule N-cadherin was found. Mechanistically, N-cadherin promoted the synaptic localization of overexpressed Neuroligin1 (Stan et al., 2010; Aiga et al., 2011; van Stegen et al., 2017).

In contrast to Neuroligin1, the properties of the synaptogenic activities of other synaptic adhesion molecules such as LRRTM2 have not been well studied. Recently, a report comparing the synaptogenic activities of Neuroligin1 and LRRTM2 in non-neuronal HEK293 cells suggested that the synaptogenic activity of LRRTM2 also depends on N-cadherin (Yamagata et al., 2018). However, the characteristics of the synaptogenic activity of LRRTM2 might be rather different upon overexpression in the specialized molecular context of neurons. In this study, we now overexpressed Neuroligin1 and LRRTM2 in cultured mouse cortical neurons and characterized their synaptogenic activities. We found that the properties of the synaptogenic activities of Neuroligin1 and LRRTM2 are rather different in cortical neurons. The synaptogenic activity of Neuroligin1 was strongly dependent on developmental maturation *in vitro*, whereas the synaptogenic activity of LRRTM2 was not. Most interestingly, while the synaptogenic activity of Neuroligin1 was dependent on N-cadherin, the synaptogenic activity of LRRTM2 was completely independent of N-cadherin expression and function in neurons.

## Methods

Methods were carried out in accordance with all relevant guidelines.

### Cell Culture

Primary neuronal mass cultures were prepared from cortices of C57/BL6 wild type mice and from Ncad^flox/flox^ mice (Kostetskii et al., 2005) as described previously (Andreyeva et al., 2012; van Stegen et al., 2017). Briefly, cortices from E18 fetuses were isolated, cut into pieces, and these pieces were trypsinized for 5 min. Brain tissue was mechanically triturated in Basal Medium Eagle (BME) media (Gibco) after removal of trypsin, and then centrifuged to get a cell pellet. The pellet was resuspended in fresh BME and 20,000–30,000 neurons were seeded on poly-L-ornithine coated glass cover slips. Fresh neurobasal (NB) medium (Gibco) with B27 supplement (2%, 50x; Gibco) containing penicillin-streptomycin (Gibco) and Glutamax-1 (Gibco) was added to the cells after 2 h. These mass cultures were maintained in a humidified incubator with 5% CO_2_ at 37°C for 7–14 days *in vitro* (DIV).

### Transfection and Plasmids

Cultured neurons were transfected by using magnetic nanoparticles (NeuroMag; OZ Biosciences) as described previously (van Stegen et al., 2017). In brief, plasmid DNA and NeuroMag were mixed in NB medium without any supplement, and incubated for 20 min at room temperature to form complexes. These complexes were directly added to the cultures, and an oscillating magnetic field was applied by placing the cultures (6-well plate) on a magnetic plate (Magnetofection^TM^, magnefect LT; nanoTherics) to enhance the transfection efficiency. After transfection, 500 μl fresh culture medium was added. The following plasmids were used: pEGFP-N1 (Clontech), Neuroligin1-EGFP (gift from Dr. T. Dresbach, Göttingen, Germany), myc-LRRTM2 (gift from Dr. J. de Wit, Leuven, Belgium), pBS598EF1alpha-EGFPcre (Addgene), and pcDNA3.1-FLAG-NCadCTF1 (ΔE N-cadherin expression plasmid; Andreyeva et al., 2012). For conditional N-cadherin knockout experiments, mass cultures from Ncad^flox/flox^ mice (Kostetskii et al., 2005) were co-transfected with EGFP, Cre-EGFP plasmid, and the respective cell adhesion molecule. For the other experiments, synaptic adhesion molecules were co-transfected with EGFP in mass cultures from wild type mice.

### Immunocytochemistry and Antibodies

Standard immunocytochemical stainings were performed 2–5 days after transfection as described previously (Nieweg et al., 2015). Cultures on coverslips were fixed for 20 min with 4% paraformaldehyde (PFA) and rinsed with phosphate-buffered saline (PBS) to remove excess PFA. After fixation, cells were permeabilized with 0.3% Triton X-100 in blocking buffer [10% fetal bovine serum (FBS), 5% sucrose, 2% bovine serum albumin (BSA), in PBS, pH 7.4] for 30 min at room temperature. Cultures were then incubated for 1 h with primary antibodies, followed by washing 3 × 10 min with PBS. Alexa Fluor (AF) conjugated secondary antibodies were added for 1–2 h at room temperature. Samples were again rinsed 3 × 10 min with PBS, and coverslips were mounted to reduce photobleaching. The following primary antibodies were used: anti-VAMP2 (rabbit polyclonal, 1:2000, Abcam Cat.No.3347), anti-VGLUT1 (guinea pig polyclonal, 1:1000, Synaptic Systems Cat.No.135304), anti-Homer1 (rabbit polyclonal, 1:1000, Synaptic Systems Cat.No.16003), anti-VGAT (mouse monoclonal, 1:1000, Synaptic Systems Cat.No.131011), anti-Gephyrin (rabbit monoclonal, 1:1000, Synaptic Systems Cat.No.147008), anti-Myc tag (mouse monoclonal, 1:1000, Thermo Fischer Cat.No.R950-25). The secondary antibodies used were: AF 555 (goat anti-rabbit, A-21428; Life Technologies), AF 555 (goat anti-mouse, A-21424; Life Technologies), AF 647 (goat anti-mouse, 115-605-003; Jackson ImmunoResearch), AF 647 (goat anti-guinea pig, 106-605-003; Jackson ImmunoResearch).

### Fluorescence Imaging and Data Analysis

Wide-field fluorescence imaging was performed using an inverted motorized Axiovert 200 M microscope (Zeiss) with 40x/1.3 oil objective as described previously (Nieweg et al., 2015; van Stegen et al., 2017). In brief, fluorescence images were captured with a 12 bit CoolSnap ES2 CCD camera (Photometrics) using MetaVue or VisiView software (Molecular Devices/Visitron Systems). The following filter sets (Zeiss) were used: (1) for EGFP (dendrites): excitation 485/20 nm, beam splitter 510 nm, emission 515/565 nm; (2) for AF 555 (VAMP2, Homer1, Gephyrin, Myc): excitation 545/25 nm, beam splitter 570 nm, emission 605/70 nm; (3) for AF 647 (VGAT, VGLUT1): excitation 640/30 nm, beam splitter 660, emission 690/50. For data analysis, images were thresholded and further processed offline using MetaMorph software (Molecular Devices/Visitron Systems). After thresholding, a low pass filter was applied to exclude single pixel noise. The processed images of immunostainings were given preudo colors before making overlay images. The overlay image was then used to identify immunopositive puncta located on the dendrites of transfected neurons. For VAMP2 analysis puncta number on dendrites was counted by creating user defined regions of interest around VAMP2 puncta and puncta density was calculated as puncta/10 μm dendrite length. Glutamatergic and GABAergic synapses were identified by colocalization of either VGLUT1 + Homer1 or VGAT + Gephyrin. To calculate puncta density, synapses on the dendrites of transfected neurons were counted. In case of GABAergic synapses, somatic synapses were also included.

### Statistics

All data are given as individual values, and as means ± SEM. Statistical significance was determined by Student’s *t*-test, if applicable, and by one-way ANOVA in combination with the Holm–Sidak *post hoc* test or by one-way ANOVA on Ranks with Dunn’s method by using SigmaPlot 11 software.

## Results

### Differential Developmental Maturation of the Synaptogenic Activities of Neuroligin1 and LRRTM2

**FIGURE 1:**
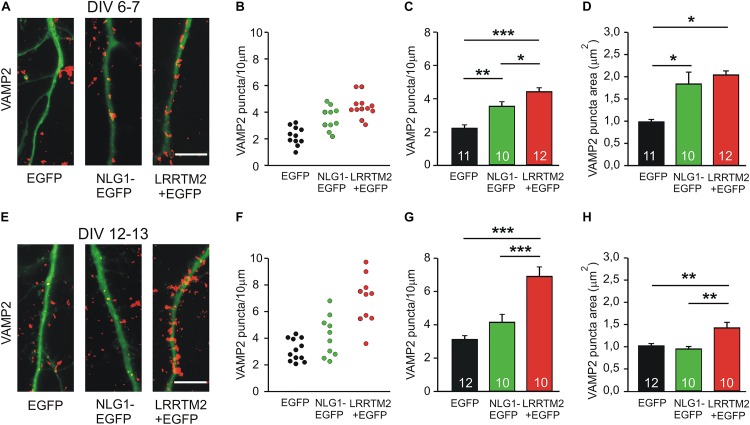
Synaptogenic activities of Neuroligin1 and LRRTM2 at different stages of neuronal differentiation *in vitro*. **(A–D)** Synaptogenic activities in cultured cortical neurons at 6–7 days *in vitro* (DIV). **(A)** Example overlay images of VAMP2-immunostained puncta (red) on dendrites visualized by EGFP expression (green; NLG1-EGFP fusion protein; LRRTM2 + EGFP co-expression). Scale bar: 10 μm. **(B)** VAMP2-positive puncta density on dendrites in individual neurons. **(C)** Mean VAMP2-positive puncta density on dendrites (*n* = 11/10/12). **(D)** Mean VAMP2 puncta area. **(E–H)** Synaptogenic activities in cultured cortical neurons at 12–13 DIV. **(E)** Example overlay images of VAMP2-immunostained puncta (red) on dendrites visualized by EGFP expression (green). Scale bar: 10 μm. **(F)** VAMP2-positive puncta density on dendrites in individual neurons. **(G)** Mean VAMP2-positive puncta density on dendrites (*n* = 12/10/10). **(H)** Mean VAMP2 puncta area. Note the loss of the synaptogenic activity of Neuroligin1 with neuronal differentiation. ^∗^ *P* < 0.05, ^∗∗^ *P* < 0.01, ^∗∗∗^ *P* < 0.001, **(C,G,H)**: one-way ANOVA with Holm–Sidak *post hoc* test, **(D)**: one-way ANOVA on Ranks with Dunn’s method.

As a first step in comparing the synaptogenic activities of Neuroligin1 and LRRTM2, we studied the developmental maturation of the synaptogenic activity of Neuroligin1 and LRRTM2 in cultured mouse cortical neurons. We analyzed the induction of presynaptic vesicle clusters by postsynaptic overexpression of Neuroligin1 and LRRTM2, respectively in individual neurons at different stages of *in vitro* differentiation. Presynaptic vesicle clusters on dendrites were immunocytochemically stained for the synaptic vesicle protein VAMP2 and dendrites were visualized by EGFP fluorescence (EGFP fusion protein/co-transfection) (Figures 1A,E). At 6–7 days *in vitro* (DIV), we observed a significant increase in the dendritic density of VAMP2-positive puncta (presynaptic vesicle clusters) upon overexpression of Neuroligin1-EGFP (transfection at 4 DIV; Figures 1B,C). This indicates a strong synaptogenic effect of Neuroligin1 in immature neurons. Overexpression of LRRTM2 led to a similar significant increase in the dendritic density of VAMP2-positive puncta indicating a comparable synaptogenic activity. In addition, significant increases in VAMP2 puncta areas were also observed (Figure 1D). The overexpression of LRRTM2 in EGFP-labeled transfected neurons was confirmed by antibody-staining of the myc-tag attached to the LRRTM2 construct (Supplementary Figure S1). Intriguingly, at 12–13 DIV we did not find a significant change in the dendritic density and in the area of VAMP2-positive puncta upon overexpression of Neuroligin1-EGFP (transfection at 9 DIV; Figures 1F–H). This suggests a developmental loss of the synaptogenic activity of Neuroligin1. In contrast, overexpression of LRRTM2 revealed a strong synaptogenic activity at 12–13 DIV. In summary, our results demonstrate that there is a differential developmental regulation of the synaptogenic activities of Neuroligin1 and LRRTM2. Neuroligin1 exhibited a strong downregulation of its synaptogenic activity during neuronal maturation, whereas LRRTM2 appeared to be effective independent of neuronal development.

**FIGURE 2:**
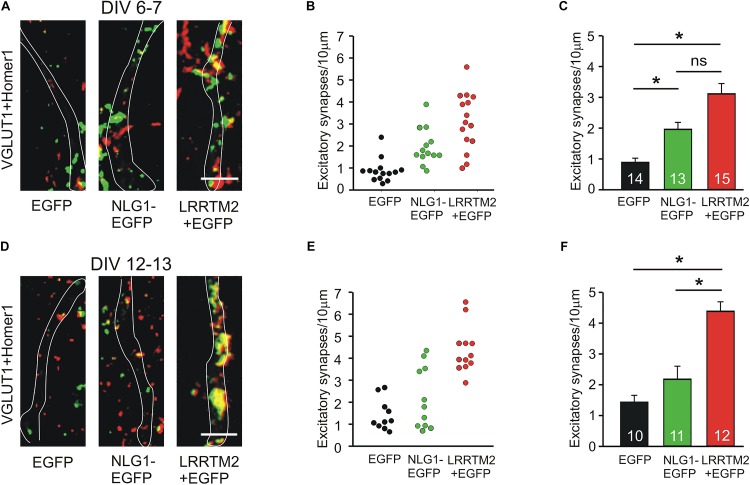
Induction of excitatory synapses by Neuroligin1 and LRRTM2 at different stages of neuronal differentiation *in vitro*. **(A–C)** Induction of excitatory synapses in cultured cortical neurons at 6–7 days *in vitro* (DIV). **(A)** Example overlay images of VGLUT1- (green) and Homer1- (red) immunostained puncta on dendrites visualized by EGFP expression (white outlines; NLG1-EGFP fusion protein; LRRTM2 + EGFP co-expression). Scale bar: 5 μm. **(B)** Density of excitatory synapses (co-localizations of VGLUT1- and Homer1-positive puncta) on dendrites in individual neurons. **(C)** Mean excitatory synapse density on dendrites (*n* = 14/13/15). **(D–F)** Induction of excitatory synapses in cultured cortical neurons at 12–13 DIV. **(D)** Example overlay images of VGLUT1- (green) and Homer1- (red) immunostained puncta on dendrites visualized by EGFP expression (white outlines). Scale bar: 5 μm. **(E)** Density of excitatory synapses on dendrites in individual neurons. **(F)** Mean excitatory synapse density on dendrites (*n* = 10/11/12). Note the loss of induction of excitatory synapses by Neuroligin1 with neuronal differentiation. ^∗^ *P* < 0.05, **(C,F)**: one-way ANOVA on Ranks with Dunn’s method.

**FIGURE 3:**
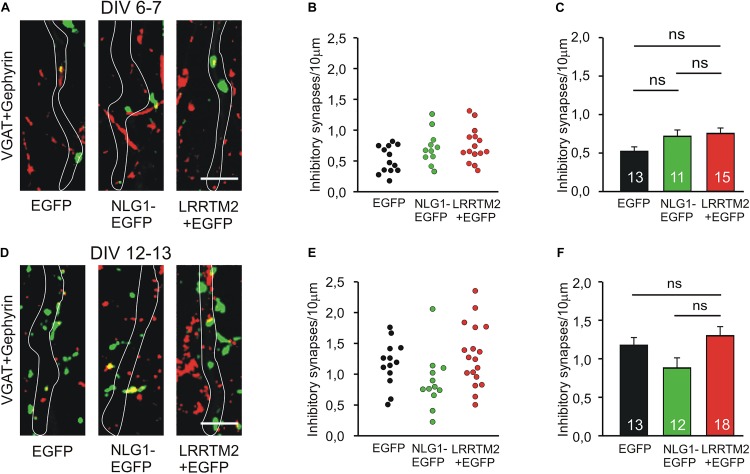
Inhibitory synapses are not induced by Neuroligin1 and LRRTM2 at different stages of neuronal differentiation *in vitro*. **(A–C)** Inhibitory synapses in cultured cortical neurons at 6–7 days *in vitro* (DIV). **(A)** Example overlay images of VGAT- (green) and Gephyrin- (red) immunostained puncta on dendrites visualized by EGFP expression (white outlines; NLG1-EGFP fusion protein; LRRTM2 + EGFP co-expression). Scale bar: 5 μm. **(B)** Density of inhibitory synapses (co-localizations of VGAT- and Gephyrin-positive puncta) on soma and dendrites in individual neurons. **(C)** Mean inhibitory synapse density on soma and dendrites (*n* = 13/11/15). **(D–F)** Inhibitory synapses in cultured cortical neurons at 12–13 DIV. **(D)** Example overlay images of VGAT- (green) and Gephyrin- (red) immunostained puncta on dendrites visualized by EGFP expression (white outlines). Scale bar: 5 μm. **(E)** Density of inhibitory synapses on soma and dendrites in individual neurons. **(F)** Mean inhibitory synapse density on soma and dendrites (*n* = 13/12/18). ns: non-significant, **(C,F)**: one-way ANOVA with Holm–Sidak *post hoc* test.

We next studied the induction of specific types of synapses (glutamatergic and GABAergic synapses) by the synaptogenic activity of overexpressed Neuroligin1 and LRRTM2, respectively. Glutamatergic synapses were visualized as co-localizations of VGLUT1 immunostained puncta (presynaptic vesicles) with Homer1 immunostained puncta (postsynaptic density) on EGFP labeled dendrites of transfected cultured mouse cortical neurons. At 6–7 DIV (transfection at 4 DIV), we observed a significant increase in the dendritic density of VGLUT1/Homer1 co-localizations upon overexpression of Neuroligin1-EGFP (Figures 2A–C). This indicates an induction of glutamatergic synapses in immature neurons. Overexpression of LRRTM2 led to a similar significant increase in the dendritic density of VGLUT1/Homer1 co-localizations indicating a comparable glutamatergic synapse induction. Intriguingly, at 12–13 DIV (transfection at 9 DIV) we did not find a significant change in the dendritic density of glutamatergic synapses upon overexpression of Neuroligin1-EGFP (Figures 2D–F), confirming a developmental loss of the synaptogenic activity of Neuroligin1. In contrast, overexpression of LRRTM2 revealed a strong induction of glutamatergic synapses also in more mature neurons. These results again demonstrate that there is a differential developmental regulation of the induction of glutamatergic synapses by Neuroligin1 and LRRTM2, respectively. In addition, GABAergic synapses were visualized by co-localizations of VGAT immunostained puncta (presynaptic vesicles) with gephyrin immunostained puncta (postsynaptic scaffold) on the soma and the dendrites (labeled by EGFP) of transfected neurons. At 6–7 DIV (transfection at 4 DIV), we did not observe any significant increase in the somatodendritic density of GABAergic synapses upon overexpression of Neuroligin1-EGFP or LRRTM2 (Figures 3A–C). Similarly, at 12–13 DIV (transfection at 9 DIV) we did not detect a significant increase in the somatodendritic density of GABAergic synapses (Figures 3D–F), indicating that overexpression of Neuroligin1-EGFP or LRRTM2 does not induce GABAergic synapses.

### Differential N-Cadherin Dependence of the Synaptogenic Activities of Neuroligin1 and LRRTM2

**FIGURE 4:**
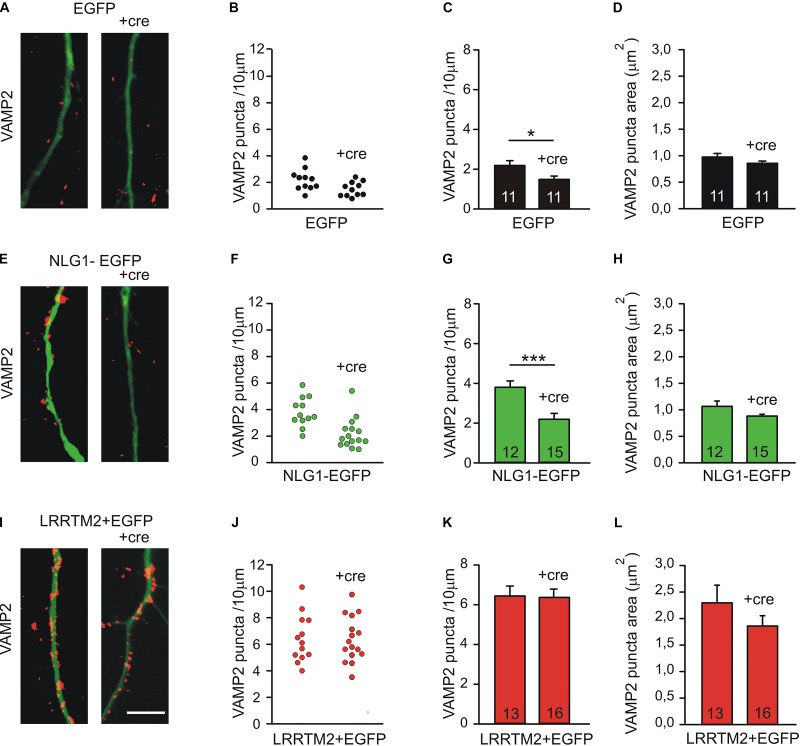
Differential dependence of the synaptogenic activities of Neuroligin1 and LRRTM2 on N-cadherin expression in immature neurons (9 DIV). **(A–D)** VAMP2-immunostained puncta density on dendrites is slightly reduced upon conditional knockout of N-cadherin by Cre expression (+cre) in individual neurons at 9 DIV. **(A)** Example overlay images (VAMP2 puncta red; EGFP expressing dendrites green). **(B)** VAMP2 puncta density on dendrites in individual neurons. **(C)** Mean VAMP2 puncta density on dendrites. *n* = 11/11. **(D)** Mean VAMP2 puncta area. **(E–H)** The enhanced density of VAMP2-immunostained puncta on dendrites upon Neuroligin1 expression (synaptogenic activity) is strongly reduced in N-cadherin knockout neurons (+cre). **(E)** Example overlay images (VAMP2 puncta red; EGFP expressing dendrites green). **(F)** VAMP2 puncta density on dendrites in individual neurons. **(G)** Mean VAMP2 puncta density on dendrites. *n* = 12/15. **(H)** Mean VAMP2 puncta area. **(I–L)** The enhanced density of VAMP2-immunostained puncta on dendrites upon LRRTM2 expression (synaptogenic activity) is not affected in N-cadherin knockout neurons (+cre). **(I)** Example overlay images (VAMP2 puncta red; EGFP expressing dendrites green). **(J)** VAMP2 puncta density on dendrites in individual neurons. **(K)** Mean VAMP2 puncta density on dendrites. *n* = 13/16. **(L)** Mean VAMP2 puncta area. Scale bar: 10 μm. ^∗^ *P* < 0.05, ^∗∗∗^ *P* < 0.001, Student’s *t*-test.

To further compare the synaptogenic activities of Neuroligin1 and LRRTM2, we investigated the dependence of the synaptogenic activity of Neuroligin1 and LRRTM2 on N-cadherin expression in cultured mouse cortical neurons. To induce a conditional knockout of N-cadherin, individual cultured cortical neurons from floxed N-cadherin mice (Kostetskii et al., 2005) were transfected with a cre expression vector (cre-EGFP plasmid) at 4 DIV. This scarce expression resulted in a mainly postsynaptic knockout of N-cadherin. To study the dependence of the synaptogenic activities on N-cadherin expression, we co-transfected Neuroligin1-EGFP and LRRTM2 (+EGFP), respectively, in these immature neurons. The analysis of synaptogenic activities was done 5 days later to enable a sufficient knockdown of N-cadherin at the protein level (Pielarski et al., 2013). In immature neurons at 9 DIV, presynaptic vesicle clusters on dendrites were immunocytochemically stained for the synaptic vesicle protein VAMP2. Dendrites of transfected neurons were visualized by EGFP fluorescence. In line with our previous reports (Stan et al., 2010; van Stegen et al., 2017), knockdown of N-cadherin expression without any overexpression resulted in a slightly reduced dendritic density of VAMP2-positive puncta (Figures 4A–D). As expected, overexpression of Neuroligin1 resulted in an increase in dendritic density of VAMP2-positive puncta, which was strongly inhibited upon conditional knockout of N-cadherin (Figures 4E–H). Overexpression of LRRTM2 also resulted in an increase in dendritic density and area of VAMP2-positive puncta, which, however, was not inhibited upon conditional knockout of N-cadherin (Figures 4I–L). In summary, our results confirm that the synaptogenic activity of Neuroligin1 in immature neurons is strongly dependent on N-cadherin expression. Most intriguingly, the synaptogenic activity of LRRTM2 was independent of N-cadherin expression in immature neurons indicating a differential regulation of the synaptogenic activities of Neuroligin1 and LRRTM2.

**FIGURE 5:**
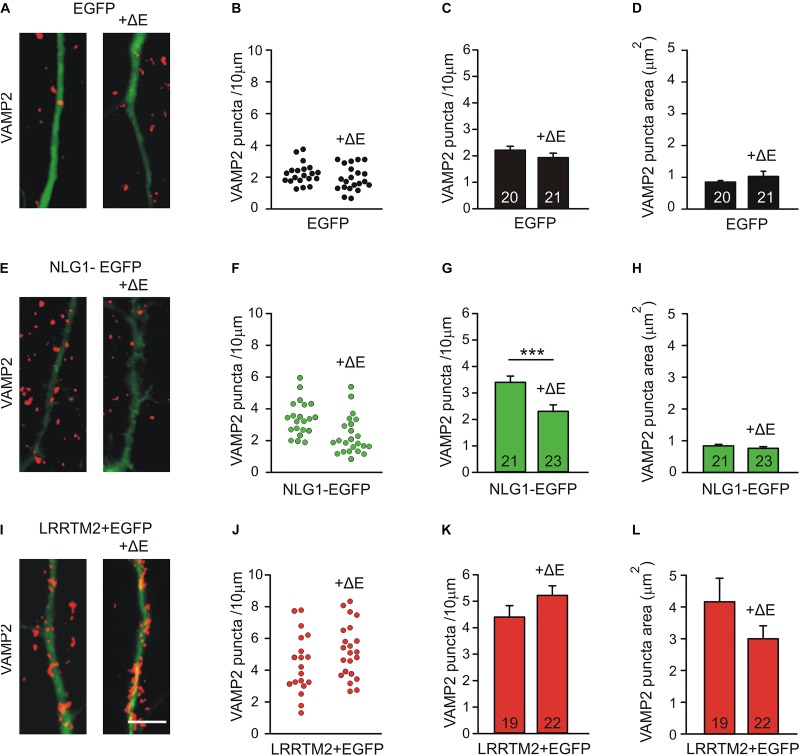
Differential dependence of the synaptogenic activities of Neuroligin1 and LRRTM2 on N-cadherin function in immature neurons (7 DIV). N-cadherin function was inhibited by expressing a dominant-negative N-cadherin mutant protein with deleted extracellular domains (+ΔE). **(A–D)** VAMP2-immunostained puncta density on dendrites is slightly reduced upon blocking N-cadherin function (+ΔE) in individual neurons at 7 DIV. **(A)** Example overlay images (VAMP2 puncta red; EGFP expressing dendrites green). **(B)** VAMP2 puncta density on dendrites in individual neurons. **(C)** Mean VAMP2 puncta density on dendrites. *n* = 20/21. **(D)** Mean VAMP2 puncta area. **(E–H)** The enhanced density of VAMP2-immunostained puncta on dendrites upon Neuroligin1 expression (synaptogenic activity) is significantly reduced upon blocking N-cadherin function (+ΔE). **(E)** Example overlay images (VAMP2 puncta red; EGFP expressing dendrites green). **(F)** VAMP2 puncta density on dendrites in individual neurons. **(G)** Mean VAMP2 puncta density on dendrites. *n* = 21/23. **(H)** Mean VAMP2 puncta area. **(I–L)** The enhanced density of VAMP2-immunostained puncta on dendrites upon LRRTM2 expression (synaptogenic activity) is not affected upon blocking N-cadherin function (+ΔE). **(I)** Example overlay images (VAMP2 puncta red; EGFP expressing dendrites green). **(J)** VAMP2 puncta density on dendrites in individual neurons. **(K)** Mean VAMP2 puncta density on dendrites. *n* = 19/22. **(L)** Mean VAMP2 puncta area. Scale bar: 5 μm. ^∗∗∗^ *P* < 0.001, Student’s *t*-test.

As an alternative experimental approach, we next inhibited N-cadherin function by overexpressing a dominant-negative C-terminal fragment (CTF1) of N-cadherin with the extracellular domains deleted (ΔE N-cadherin; Togashi et al., 2002; Andreyeva et al., 2012) in a few individual neurons (mainly postsynaptic overexpression) at 4 DIV. The analysis of synaptogenic activities was done at 7 DIV after 3 days of co-expression of Neuroligin1-EGFP and LRRTM2 (+EGFP), respectively. Inhibition of N-cadherin function without any further overexpression led to a slight reduction in the dendritic density of VAMP2-positive puncta (Figures 5A–D). As expected, overexpression of Neuroligin1 again resulted in an increased dendritic density of VAMP2-positive puncta that was significantly reduced upon inhibition of N-cadherin function by co-expression of ΔE N-cadherin (Figures 5E–H). Overexpression of LRRTM2 also led to an increase in dendritic density and area of VAMP2-positive puncta, which was not affected by inhibition of N-cadherin function (Figures 5I–L). Taken together, our results based on inhibition of N-cadherin function again demonstrate a differential dependence of the synaptogenic activities of Neuroligin1 and LRRTM2 on N-cadherin in immature neurons.

**FIGURE 6:**
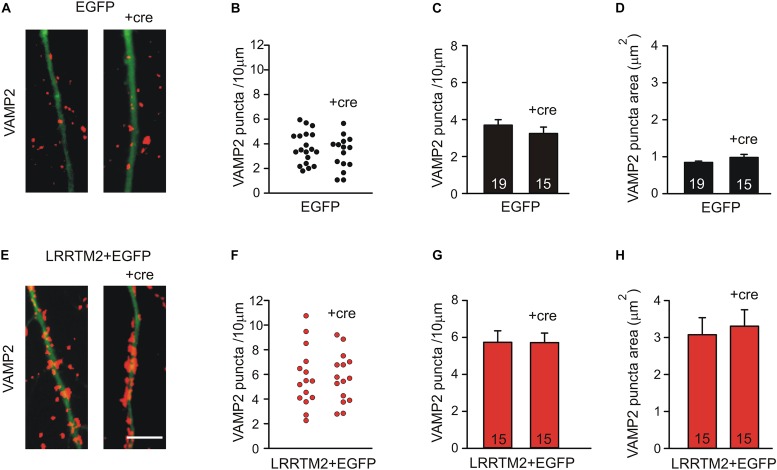
The synaptogenic activity of LRRTM2 at 14–15 DIV does not depend on N-cadherin expression. **(A–D)** VAMP2-immunostained puncta density on dendrites is not significantly altered upon conditional knockout of N-cadherin by Cre expression (+cre) in individual neurons at 14–15 DIV in control cells. **(A)** Example overlay images (VAMP2 puncta red; EGFP expressing dendrites green). **(B)** VAMP2 puncta density on dendrites in individual neurons. **(C)** Mean VAMP2 puncta density on dendrites. *n* = 19/15. **(D)** Mean VAMP2 puncta area. **(E–H)** The enhanced density of VAMP2-immunostained puncta on dendrites upon LRRTM2 expression (synaptogenic activity) is not affected in N-cadherin knockout neurons (+cre). **(E)** Example overlay images (VAMP2 puncta red; EGFP expressing dendrites green). **(F)** VAMP2 puncta density on dendrites in individual neurons. **(G)** Mean VAMP2 puncta density on dendrites. *n* = 15/15. **(H)** Mean VAMP2 puncta area. Scale bar: 5 μm.

To further corroborate that the synaptogenic activity of LRRTM2 in cortical neurons is independent of N-cadherin expression, we studied overexpression of LRRTM2 in more mature neurons at 14 DIV, in which Neuroligin1 does not anymore exhibit a synaptogenic activity (see Figure 1). We again induced a conditional knockout of N-cadherin by transfecting a cre-EGFP expression vector at 9 DIV (co-transfection with LRRTM2 + EGFP). Analysis of the synaptogenic activity of LRRTM2 overexpression was done 5 days later to allow for sufficient knockdown of N-cadherin protein. As expected, overexpression of LRRTM2 again resulted in an increased dendritic density and area of VAMP2-positive puncta in more mature neurons. However, this synaptogenic activity of LRRTM2 was not affected by conditional knockout of N-cadherin (Figure 6). This result again demonstrates that the synaptogenic activity of LRRTM2 is independent of N-cadherin expression in cortical neurons.

## Discussion

In this paper, we compared the synaptogenic activities of the postsynaptic adhesion molecules Neuroligin1 and LRRTM2 upon overexpression in cultured mouse cortical neurons. As expected from previous reports, the synaptogenic activities of both neurexin ligands resulted in the formation of additional presynaptic vesicle clusters. This was accompanied by an induction of glutamatergic, but not GABAergic synapses. We found that the synaptogenic activity of Neuroligin1 was strongly dependent on the maturational state of neurons and was lost with ongoing neuronal differentiation *in vitro*. In contrast, the synaptogenic activity of LRRTM2 was not altered during neuronal maturation in culture, strongly indicating that the properties of the synaptogenic activities of both adhesion molecules are rather different in the molecular context of neurons. Furthermore, we observed that the synaptogenic activity of Neuroligin1 is crucially dependent on the expression and function of the adhesion molecule N-cadherin, whereas the synaptogenic activity of LRRTM2 did not require N-cadherin expression or function. These results demonstrate that the requirements to exhibit synaptogenic activity in neurons are rather different for Neuroligin1 and LRRTM2.

The dependence of the synaptogenic activity of Neuroligin1 on N-cadherin has been described previously (Stan et al., 2010; Aiga et al., 2011). Also the molecular mechanism consisting of a direct interaction of both Neuroligin1 and the N-cadherin/β-cateinin complex with specific domains of the postsynaptic scaffolding molecule S-SCAM has been elucidated (Stan et al., 2010; Aiga et al., 2011). In this paper, the synaptogenic activity of Neuroligin1 served as a positive control for analyzing the properties of the synaptogenic activity of LRRTM2.

Seemingly contrary to our findings, Yamagata et al. (2018) recently described in a presynaptic neuron and non-neuronal HEK293 cell co-culture system that the synaptogenic activities of both Neuroligin1 and LRRTM2 depend on N-cadherin expression. However, in this non-physiological co-culture system also branching and contact formation of presynaptic axons with HEK293 cells was strongly dependent on the adhesion molecule N-cadherin. Because intercellular contact formation appears to be a crucial requirement for synaptogenic activity in presynaptic axons, a defect in contact formation will lead to an apparent loss of synaptogenic activity (Yamagata et al., 2018). At our neuron–neuron contacts, other adhesion molecules might compensate for the loss of the intercellular adhesive function of N-cadherin (Jüngling et al., 2006).

Synaptic cell adhesion proteins perform their functional roles in the context of a large number of other pre- and postsynaptic proteins. Therefore, the cellular context, e.g., HEK293 cells versus cortical neurons, in which they are expressed may have a crucial influence on the observed functional properties. The lateral mobility of adhesion molecules at synaptic and extrasynaptic sites is among the most basic regulatory influences exerted by cellular context proteins, e.g., by postsynaptic scaffolding molecules (Chamma et al., 2019). Neuroligin1 has been described by single-molecule nanoscale imaging to be rather dispersed and highly dynamic at dendritic spines. By contrast, LRRTM2 is organized in compact and stable synaptic nanodomains (Chamma et al., 2016a, b). The N-cadherin adhesion system might strongly modulate the high lateral mobility of Neuroligin1 thereby potentially enhancing its synaptic localization, whereas LRRTM2 might be subsynaptically anchored by other molecular mechanisms and thus might not require N-cadherin for synaptic localization. This might explain the differential requirement for N-cadherin expression of the synaptogenic activities of Neuroligin1 and LRRTM2, respectively. In this paper, we aimed at comparing the synaptogenic activities of LRRTM2 and Neuroligin1 in *in vitro* cell culture systems. *In vivo* gene knockout and knockdown studies indicated that the physiological functions of LRRTM2 and Neuroligin1 seem to be different from their *in vitro* synaptogenic activities. *In vivo*, specific functions in modulating synaptic transmission and enabling long-term potentiation have been described (Varoqueaux et al., 2006; Soler-Llavina et al., 2011, 2013; Jiang et al., 2017; Bhouri et al., 2018).

In conclusion, our analysis of the properties of the synaptogenic activities of the postsynaptic adhesion molecules Neuroligin1 and LRRTM2 revealed strong differences between these two neurexin ligands. A basic molecular difference between Neuroligin1 and LRRTM2 in lateral mobility at neuronal synaptic sites (Chamma and Thoumine, 2018) might well explain their differential requirements for exhibiting synaptogenic activity.

## Data Availability Statement

The datasets generated for this study are available on request to the corresponding author.

## Ethics Statement

The animal study was reviewed and approved by Tierschutzbeauftragte, Heinrich Heine University Düsseldorf.

## Author Contributions

SD performed experiments, analyzed data, and wrote the manuscript. KG designed research and wrote the manuscript.

## Conflict of Interest

The authors declare that the research was conducted in the absence of any commercial or financial relationships that could be construed as a potential conflict of interest.

## Supplementary Material

FIGURE S1 Confirmation of the overexpression of LRRTM2 in transfected neurons. **(A)** LRRTM2 transfected neuron labeled by co-transfected EGFP (green). **(B)** Immunocytochemical staining for the myc tag (red) which had been attached to the LRRTM2 construct. **(C)** Overlay of images in **(A)** and **(B)**. Scale bar: 20 μm.
