# Supplementary figures and images for: Differential Properties of the Synaptogenic Activities of the Neurexin Ligands Neuroligin1 and LRRTM2
Source: Front Mol Neurosci. 2019 Nov 8;12:269. doi: 10.3389/fnmol.2019.00269 (PMC6856695; doi:10.3389/fnmol.2019.00269)

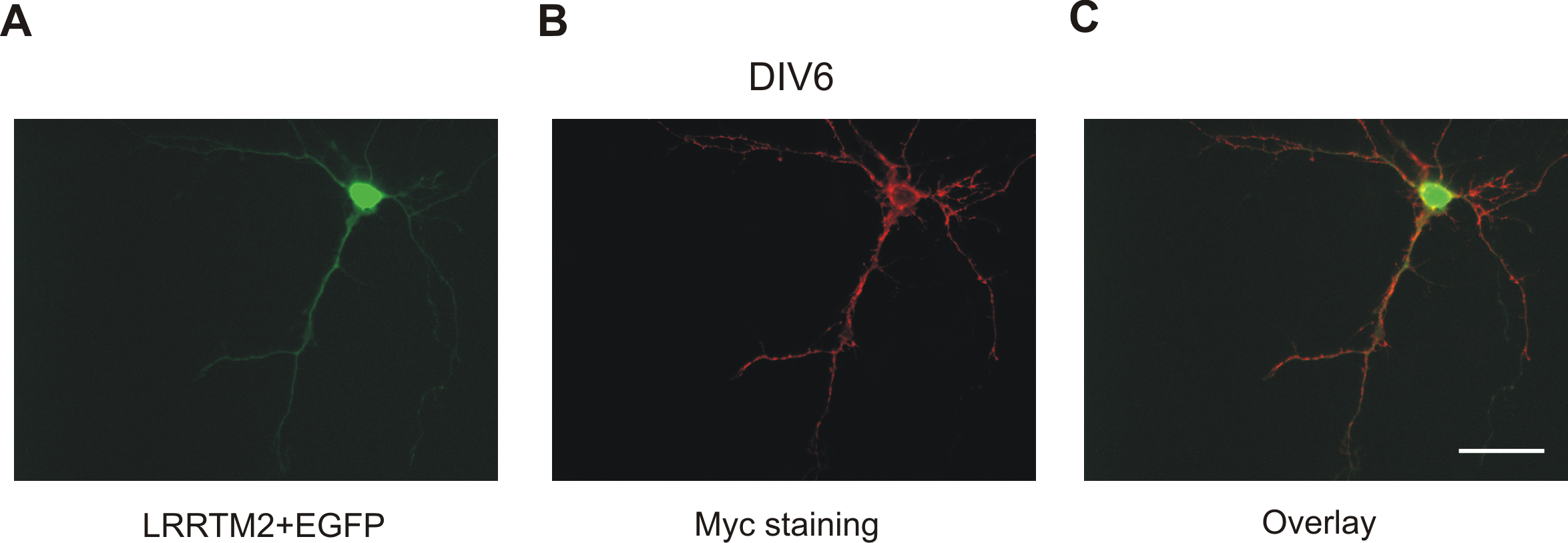

Supplement: FIGURE S1 — Confirmation of the overexpression of LRRTM2 in transfected neurons. (A) LRRTM2 transfected neuron labeled by co-transfected EGFP (green). (B) Immunocytochemical staining for the myc tag (red) which had been attached to the LRRTM2 construct. (C) Overlay of images in (A) and (B). Scale bar: 20 μm. [file Image_1.TIF]
